# A DFT Study of Volatile Organic Compounds Detection on Pristine and Pt-Decorated SnS Monolayers

**DOI:** 10.3390/s23177319

**Published:** 2023-08-22

**Authors:** Jiayin Wu, Zhongbao Li, Aiping Luo, Xiaobo Xing

**Affiliations:** 1 School of Information and Optoelectronic Science and Engineering, South China Normal University, Guangzhou 510006, China; wujiayin@m.scnu.edu.cn (J.W.); luoaiping@scnu.edu.cn (A.L.); 2 School of Materials and Chemistry Engineering, Tongren University, Tongren 554300, China; zongbaoli1982@163.com; 3 South China Academy of Advanced Optoelectronics, South China Normal University, Guangzhou 510006, China

**Keywords:** SnS monolayer, VOCs, gas sensor, two-dimensional materials, DFT, Pt-decorated, NEGF, sensitivity, first principles study, Tin monosulfide

## Abstract

Real-time monitoring of volatile organic compounds (VOCs) is crucial for both industrial production and daily life. However, the non-reactive nature of VOCs and their low concentrations pose a significant challenge for developing sensors. In this study, we investigated the adsorption behaviors of typical VOCs (C_2_H_4_, C_2_H_6_, and C_6_H_6_), on pristine and Pt-decorated SnS monolayers using density functional theory (DFT) calculations. Pristine SnS monolayers have limited charge transfer and long adsorption distances to VOC molecules, resulting in VOC insensitivity. The introduction of Pt atoms promotes charge transfer, creates new energy levels, and increases the overlap of the density of states, thereby enhancing electron excitation and improving gas sensitivity. Pt-decorated SnS monolayers exhibited high sensitivities of 241,921.7%, 35.7%, and 74.3% towards C_2_H_4_, C_2_H_6_, and C_6_H_6_, respectively. These values are 142,306.9, 23.8, and 82.6 times higher than those of pristine SnS monolayers, respectively. Moreover, the moderate adsorption energies of adsorbing C_2_H_6_ and C_6_H_6_ molecules ensure that Pt-decorated SnS monolayers possess good reversibility with a short recovery time at 298 K. When heated to 498 K, C_2_H_4_ molecules desorbs from the surface of Pt-decorated SnS monolayer in 162.33 s. Our results indicate that Pt-decorated SnS monolayers could be superior candidates for sensing VOCs with high selectivity, sensitivity, and reversibility.

## 1. Introduction

Volatile organic compounds (VOCs) are made up of a variety of components, including alkanes, alkenes, alkynes, and benzene derivatives. They are poisonous, irritating, and carcinogenic, and are distinguished by their permeability, volatility, and liposolubility. Furthermore, VOCs are precursors to a variety of other atmospheric pollutants, including secondary aerosols and chemical smog. Ethylene, ethane, and benzene are three volatile organic compounds (VOCs) with major industrial relevance but also hazardous impacts. As a plant hormone, ethylene is critical for regulating fruit ripening but is also a toxic precursor in petrochemical synthesis. Ethane is similarly an important feedstock chemical and refrigerant, yet also highly flammable. Meanwhile, the carcinogenic solvent benzene is utilized across sectors from shoemaking to pharmaceuticals, while posing risks from its rapid volatilization and human toxicity. Specifically, acute benzene poisoning via inhalation can induce neurological spasms, coma, and even death. Currently, photocatalysis [1], electrocatalysis, and photo-electrocatalysis [2] have are considered promising technologies for VOCs reduction. Plasmonic nanoparticles have emerged as promising catalysts for the degradation of volatile organic compounds (VOCs). Specifically, catalysts such as Pt-Au/graphene composites and Porous Ag_0.333_V_2_O_5_ nanorods have been developed to facilitate reactions involving methanol oxidation and oxygen reduction. Targeted VOC control solutions require real-time monitoring and detection of VOC pollution in the atmosphere to effectively prevent and control VOC pollution. However, because of their non-reactive nature and low concentrations, sensing volatile organic molecules is difficult. The classic approach for detecting VOCs is gas chromatography-mass spectrometry (GC-MS), but it needs expensive equipment and involves complex processes, making it only suited for laboratory-based gas detection.

Hence, researchers are continually attempting to build portable VOC sensors. Wencui Kang et al. fabricated a nano-colorimetric sensor array consisting of nano-chemo responsive materials, which are used to quantify the volatile alcohols (VAs) in the kombucha tea extract fermentation process [3]. Au nanoparticles decorated ZnO nanotetrapod sensors with temperature-dependent selectivity were created by Fang Xu [4]. The sensor showed higher selectivity for formaldehyde at temperatures below 200 °C, ethanol at temperatures between 200 and 340 °C, and acetone at temperatures over 400 °C. Decorating TiO_2_ gas sensors with Au nanoparticles improves sensor sensitivity by 40% according to Mostafa Shooshtari et al. [5]. At room temperature, the Au-decorated TiO_2_ sensors exhibited sensitivities of 1.1, 1.07, and 1.03 toward 50 ppm of acetone, methanol, and ethanol vapors, respectively. Despite recent advances, developing selective, reusable, and real-time VOC sensors with high responsiveness remains an urgent challenge. Of particular priority are sensors for detecting ultralow concentrations of ethylene, ethane, and benzene—critical VOC targets across diverse applications. For instance, precise detection of ethylene emission rates could enable optimal timing of fruit harvests, while ethane and benzene sensors promise significant impacts in monitoring natural gas pipelines and human exposure risks. As a result, there is an urgent need to create VOC sensors with high responsiveness, strong selectivity, reusability, and real-time monitoring.

An SnS monolayer is a two-dimensional (2D) transition metal sulfide material with an orthorhombic crystal structure and an indirect band gap of 1.63 eV [6]. An SnS monolayer has a high electron mobility of 1.93 × 10^3^ cm^−2^/Vs [7], which makes it suitable for electronic applications. Due to its moderate band gap and large absorption coefficient (>10^4^ cm^−1^), SnS monolayers can also be used as photovoltaic absorbers [8], photodetectors [9], and semiconductor devices [10]. SnS monolayers are composed of abundant and non-toxic elements in the Earth’s crust and do not contain heavy metals, which makes them low cost and eco-friendly. An SnS monolayer also exhibits a unique 2D wrinkled layer similar to black phosphorus; which provides an enlarged specific surface area and promotes gas adsorption, making it advantageous for gas sensing applications. According to Aarti et al., O_3_ and SO_3_ are chemisorbed, whereas CH_2_O, H_2_S, and HCN are physisorbed by the SnS monolayer [11]. The S-vacancy SnS monolayer was discovered to be appropriate for SO_2_ detection [12] by further work of Aarti. Hu et al. revealed that monolayer SnS is a promising candidate for use as a NO_2_ gas sensor, with physisorption properties toward NH_3_, CO, and H_2_O [13]. Because of increased conductivity and weak contact, the compressive strain (≥3%) on SnS has been demonstrated to have greater sensitivity performance to NH_3_, H_2_S, and CO by Qin. et al. [14]. Qiu et al. also created S-vacancy-enriched SnS for methanol sensing materials at ambient temperature and clarified it further using DFT calculations [15]. According to Qiu et al., Si and P doping of SnS monolayers displays higher adsorption energy and shorter recovery time to NH_3_ and SO_2_ at normal temperatures, respectively, but SnS monolayers with a Cl dope might be reusable NO_2_ sensors at high temperatures [16]. Qin et al. discovered that Ag-doped and Ag-functionalized SnS monolayers outperform pure SnS monolayers in ethanol sensing [17]. Qin et al. created pristine and Zn-doped SnS and discovered that Zn doping increased methanol vapor sensing performance [18]. Bai et al. created Mn-doped SnS that surpassed pure SnS in terms of acetone response and recovery time by 3.35 times [19]. In our previous work, we have demonstrated that SnS monolayers serve as highly selective, sensitive, and reusable sensors for heavy metal detection [20].

The sensing capabilities of pristine and Pt-decorated SnS monolayers (Pt@SnS) towards three typical VOCs, including ethylene (C_2_H_4_), ethane (C_2_H_6_), and benzene (C_6_H_6_), were comprehensively investigated using a combination of density functional theory (DFT) first-principles computations and the non-equilibrium Green’s function method (NEGF). Various key characteristics, including absorption energy, charge transfer, density of states (DOS), band structure, and current−voltage curves, are calculated for the VOCs adsorbed on both pristine and Pt-modified SnS monolayers. Pt-functionalized SnS monolayers exhibit exceptional gas sensing performance, including higher sensitivity than existing materials and distinctive bias-switchable selectivity toward multiple VOCs with a single device.

## 2. Calculation Methods

The Vienna Ab initio Simulation Package (VASP) was utilized for spin-polarized DFT calculations [21,22], with the Perdew−Burke−Ernzerhof parametrization (GGA-PBE) used to compute exchange-correlation energy [23]. The DFT-D3 method was employed to account for van der Waals interaction [24]. To eliminate interlayer interactions, a vacuum layer with a thickness of 20 Å was introduced parallel to the surface layer. Sampling of the Brillouin zone was performed using Monkhorst−Pack k-points with a grid size of 4 × 4 × 1. The energy cutoff was set at 400 eV, and convergence criteria of 10^−5^ eV and 0.02 eV/Å were applied for the energy and forces, respectively. The adsorption energy was calculated using the formula as follows:

(1) $$E_{ad}=E_{total}-E_{sub}-E_{gas}$$

The adsorption energy (E*_ad_*) was computed by subtracting the total energy of the gas adsorption system (E*_total_*) from the combined energies of the optimized pristine or Pt-decorated SnS monolayer (E*_sub_*) and the VOC molecule (E*_gas_*). The crystal structures and charge density differential (CDD) were visualized using VESTA 3 [25].

The current−voltage (I–V) characteristics were calculated using the nonequilibrium Green’s function method implemented in the TranSIESTA package of SIESTA [26]. The Landauer−Buttiker formula [27] was employed to compute the current, as expressed in Equation (2):

(2) $$I\left(V_{b}\right)=\frac{2e}{h}\int_{\mu_{R}}^{\mu_{L}}T\left(E,V_{b}\right)\left[f\left(E-\mu_{L}\right)-f\left(E-\mu_{R}\right)\right]$$

Calculations were made to determine the electric current (I) flowing through the contact electrode at a bias voltage (V*_b_*). μ_L_ and *μ_R_*, respectively, stand for the left and right electrodes’ electrochemical potentials. The left and right electrodes’ Fermi−Dirac distribution function was written as *f*(*E* − *μ_L_*_/*R*_). It was calculated the transmission coefficient *T*(*E*,*V_b_*) at a given energy (*E*) and bias voltage (*V_b_*).

## 3. Results and Discussion

### 3.1. Adsorption Characteristics of Pristine SnS Monolayer on VOCs

**Figure 1:**
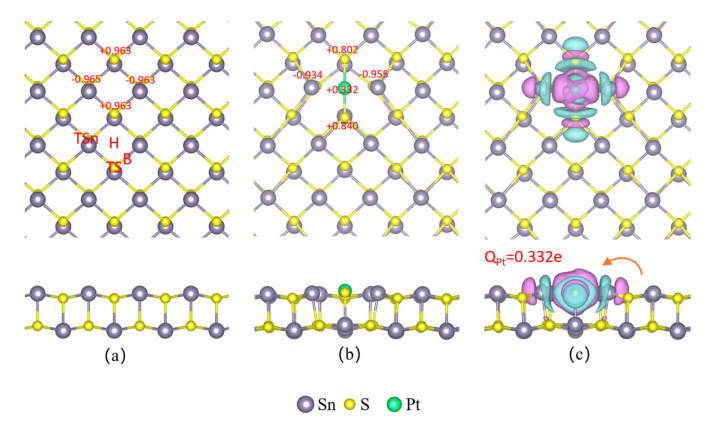
The structure and adsorption position of the SnS monolayer: (**a**) pristine SnS monolayer, (**b**) Pt-decorated SnS monolayer, (**c**) CDD of Pt-decorated SnS monolayer.

**Figure 2:**
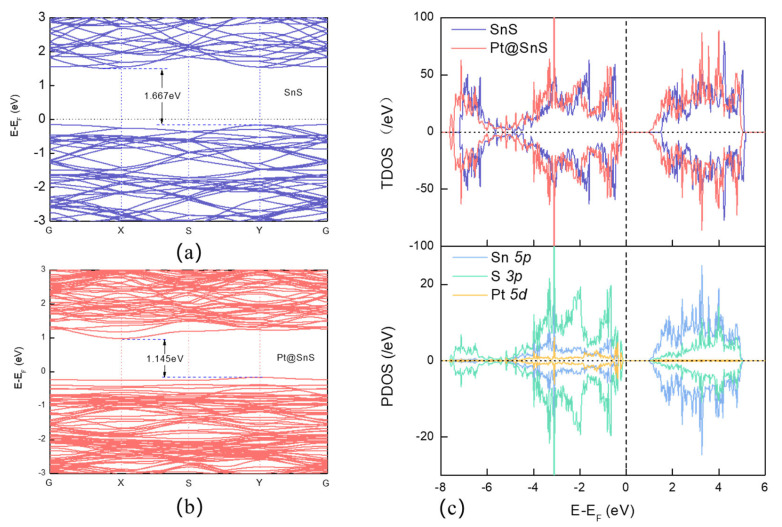
The structure and adsorption position of the SnS monolayer: (**a**) pristine SnS monolayer, (**b**) Pt-decorated SnS monolayer, (**c**) CDD of Pt-decorated SnS monolayer.

Figure 1a depicts the construction of an orthorhombic SnS monolayer using a 4 × 4 supercell (64 atoms). Each Sn(S) atom in the monolayer was coordinated with three Sn(Sn) atoms. To determine the optimal adsorption site of the SnS monolayers, four distinct locations were considered: the center of the tetragonal ring (H), the top of the Sn atom (TSn), the top of the S atom (TS), and the bridge (B) of the Sn-S bond. Figure 2a exhibits that the SnS monolayer’s bandwidth is 1.667 eV.

**Figure 3:**
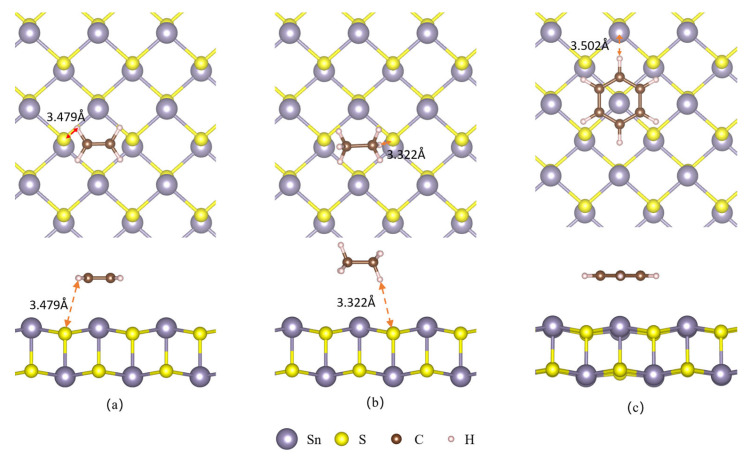
VOC adsorption on the surface of SnS monolayer. (**a**) C_2_H_4_. (**b**) C_2_H_6_. (**c**) C_6_H_6_.

Figure 3 illustrates the most stable configuration of VOC adsorption on the SnS monolayer. C_2_H_4_ and C_2_H_6_ are adsorbed at the H site of the SnS monolayer, with their C-C bonds parallel to the monolayer surface. The distance between the H atom of C_2_H_4_ and the closest S atom on the SnS monolayer surface is 3.479 Å, while that between the H atom of C_2_H_6_ and the closest S atom on the SnS monolayer surface is 3.322 Å. The hexagonal ring of C_6_H_6_ is parallel to the SnS monolayer surface, with its center located at the TSn site. The distance between the H atom of C_6_H_6_ and the Sn atom is 3.502 Å. These results reveal that the SnS monolayer exhibits weak interaction with VOC molecules due to their relatively long distance from the surface. Table 1 shows that C_2_H_4_, C_2_H_6_, and C_6_H_6_ gas molecules exhibit adsorption energies of −0.532 eV, −0.314 eV, and −0.601 eV, respectively. The adsorption of VOC molecules onto SnS monolayers is an exothermic process, as evidenced by the negative values of the adsorption energies. This implies that the adsorption can occur spontaneously, without requiring external energy input.

The CDD is computed using Equation (3) in order to further comprehend the bonding features of the adsorbate and the substrate:

(3) $$\Delta \rho =\rho_{sub+ads}-\rho_{sub}-\rho_{ads}$$

Here, the charge densities of the adsorption system, the substrate, and the adsorbent are denoted as *ρ_sub_*_+*ads*_, *ρ_sub_*, and *ρ_ads_*, respectively.

**Figure 4:**
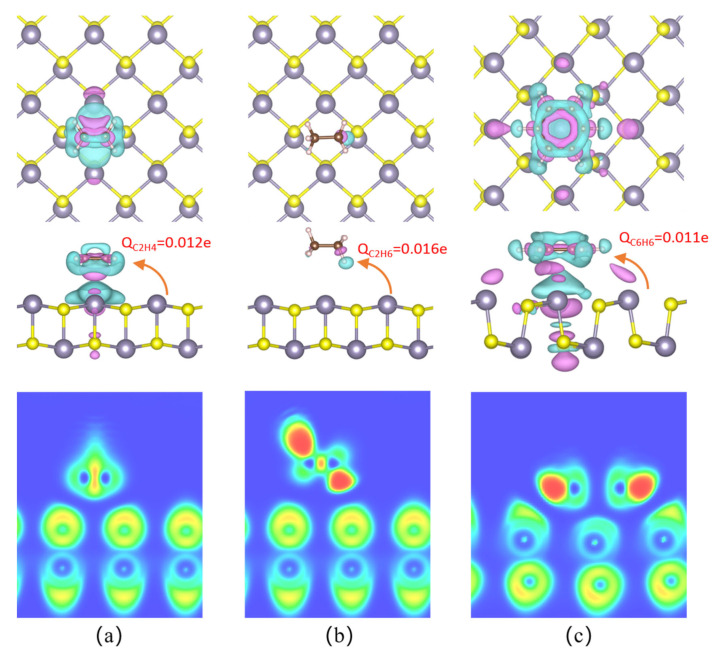
The CDD and ELF of VOC adsorption on SnS monolayers. (**a**) C_2_H_4_. (**b**) C_2_H_6_. (**c**) C_6_H_6_.

As Figure 4 shows, the charge density differential displays areas of electron accumulation in magenta and areas of electron depletion in blue. Adsorption of C_2_H_4_ onto the SnS monolayer surface creates charge depletion regions in the SnS accompanied by charge accumulation zones between the C_2_H_4_ molecule and the surface, centered on the C atoms. This redistribution indicates electron transfer from the SnS monolayer to the vicinity of the C atoms in C_2_H_4_. Bader analysis reveals that C_2_H_4_ gains 0.012 e from the SnS monolayer. For C_2_H_6_, the main charge depletion zone occurs around the H atoms near the SnS monolayer, whereas the charge accumulates on the C atoms facing the H atoms. C_2_H_6_ gains 0.016e from the SnS monolayer. In contrast, C_6_H_6_ adsorption results in charge accumulation zones on the side and bottom of the Sn atoms below C_6_H_6_ and a charge dissipation zone on the top of the Sn atoms facing C_6_H_6_. The adsorption of C_6_H_6_ results in an increased electron density on the Sn atoms and the bottom layer of the SnS monolayer, accompanied by electron depletion in the direction towards the C_6_H_6_ molecule. This signifies electron transfer from the SnS to the C_6_H_6_. Specifically, accumulation zones centered on the six C atoms indicate they gain 0.011e each from the SnS monolayer. Our simulations reveal only weak charge transfer (0.011–0.016 eV) from the SnS monolayer to the adsorbing C_2_H_4_, C_2_H_6_, and C_6_H_6_ gas molecules. This confirms that these hydrocarbon species act as electron acceptors, depleting a small fraction of electron density from the SnS during physisorption.

The electron localization function (ELF) calculations are performed as well as analyses to investigate the interactions between three gas molecules and the SnS monolayer as seen in Figure 4. The figure displays the degree of electron localization (red) and delocalization (blue) in the region. It is evident that, after adsorption, there is no electron localization overlap between gas molecules and adsorbents, indicating no chemical reaction but physical interaction.

**Figure 5:**
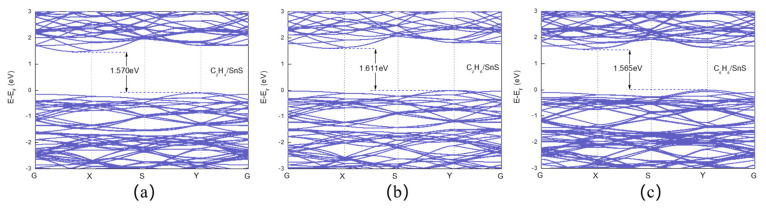
The band structures of VOC adsorption on SnS monolayers. (**a**) C_2_H_4_. (**b**) C_2_H_6_. (**c**) C_6_H_6_.

**Figure 6:**
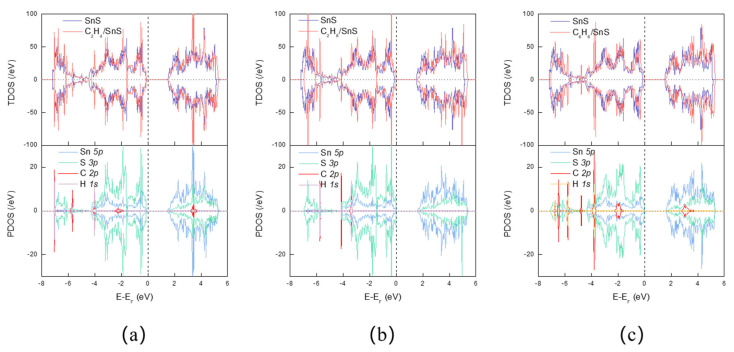
The TDOS and PDOS of VOC adsorption on SnS monolayers. (**a**) C_2_H_4_. (**b**) C_2_H_6_. (**c**) C_6_H_6_.

After VOC adsorption, the bandwidth narrows, as can be observed in Figure 5. The bandwidths of C_2_H_4_, C_2_H_6_, and C_6_H_6_ are reduced to 1.570 eV, 1.611 eV, and 1.565 eV, respectively. The TDOS and projected density of states (PDOS) of VOC adsorption on SnS monolayers are plotted in Figure 6, which illuminates that the adsorption of VOC molecules onto SnS monolayers does not result in obviously overlap between the C 2p or H 1s orbitals and Sn 5p or S 3p orbitals near the Fermi level. Consequently, hybridized energy levels do not form at the conduction and valence band edges, resulting in a limited impact on the energy bands due to gas adsorption. Moreover, after absorbing C_2_H_4_ and C_6_H_6_, the contribution of the gas’ C 2p orbitals to the peaks at 3.102 eV and 3.030 eV, respectively, is weak. Insufficient overlap between the molecular orbitals of a VOC species and the electronic states of the SnS monolayer surface suggests weak interfacial interactions. Minimal orbital overlap also implies a poor match between the relative energy levels of the molecule and monolayer. Transitions between the molecular orbitals and SnS band states are then less likely to occur. Therefore, it can be inferred that the adsorption of VOCs onto SnS monolayers has an insignificant impact on the density of states in the conduction band. This observation aligns with the finding that there is a weak interaction between VOCs and the system, as discussed earlier. DOS and band structure analyses indicate minimal perturbations to the electronic structure of the SnS monolayer upon VOC molecule adsorption, which suggests that electrical conductivity modulation will be very small in the presence of VOCs. This implies that VOC sensitivity will be low in bare SnS monolayers due to weak interfacial interactions.

**Figure 7:**
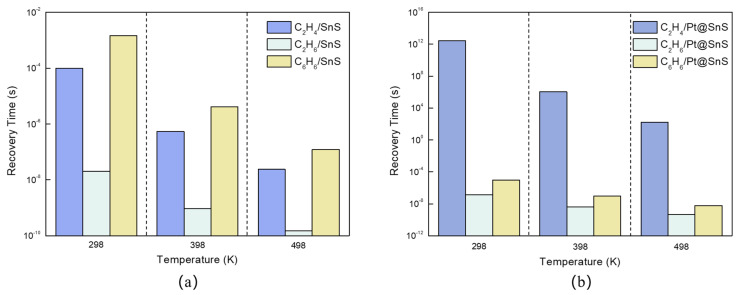
The recovery time of VOC adsorption on SnS monolayers. (**a**) Intrinsic SnS monolayer; (**b**) Pt-decorated SnS monolayers.

Recovery time is the time required for gas molecules to desorb from the surface of the sensing material. This is a key indicator of the reusability of the sensor material. The shorter the recovery time, the higher the reusability of the material, but the weaker the interaction between the material and gas molecules. For materials with a longer recovery time, the desorption process can be accelerated by increasing the temperature or applying UV irradiation [28]. Using the classical transition state theory, the recovery time (τ) is calculated with:

(4) $$\tau =\exp\left(\frac{-E_{\mathrm{ad}}}{k_{B}T}\right)/\omega$$

where *ω*, E_ad_, k_B_, and T are the attempt frequency, adsorption energy, Boltzmann constant, and temperature, respectively. *ω* is taken as 10^−13^ s^−1^ [16]. From our recovery time calculations, we find that the recovery times of C_2_H_4_, C_2_H_6_, and C_6_H_6_ at room temperature (298 K) are 9.59 × 10^−5^ s, 2.04 × 10^−8^ s, and 1.46 × 10^−3^ s, respectively, as shown in Figure 7a. The short recovery time of the pristine SnS monolayer indicates its potential for good reusability in VOC sensing applications. However, this also suggests that the selectivity and sensitivity of the sensor toward VOC molecules may be compromised.

**Figure 8:**
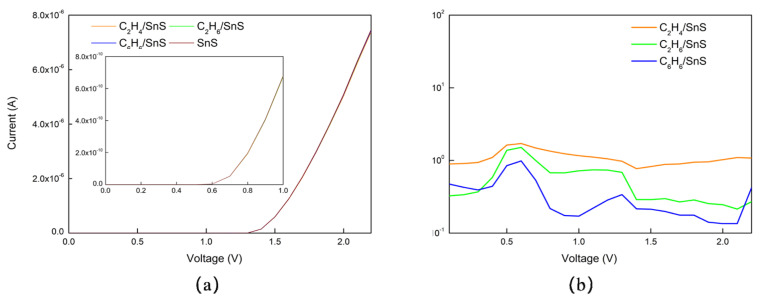
The (**a**) current−voltage curves and (**b**) sensitivity of VOC adsorption on SnS monolayer.

Leveraging NEGF theory, the electrical transport properties of SnS monolayers are investigated through comprehensive two-probe simulations to analyze the current−voltage (I–V) characteristics of SnS monolayers in detail, as apparent from Figure S1. This model consists of two electrodes and a scattering region, where the surface of the latter is capable of detecting VOC molecules. Figure 8a illustrates that the current of the SnS monolayer with adsorbed VOCs closely resembles that of the pristine SnS monolayer, making it challenging to differentiate between the current curves of different gases from the graph. This observation further suggests that the change in conductivity of the SnS monolayer after adsorbing VOCs is negligible.

To further evaluate the VOC sensing abilities of SnS monolayers, their sensitivity (S) and conductance (G) are estimated using Equations (5) and (6), respectively:

(5) $$S=\left|{G-G}_{0}\right|/G_{0}$$

(6) $$G=I/V_{b}$$

The conductivity of the SnS monolayer with and without deposited VOC molecules is represented by G and G_0_. The bias voltage applied to the electrodes is denoted by V_b_, whilst the current flowing through the scattering region with adsorbed VOC molecules is denoted by I.

As Figure 8a shows, our electronic transport simulations quantify the minor conductivity changes in the SnS monolayer upon VOC adsorption. For C_2_H_4_, the conductivity changes by 0.8–1.7% relative to bare SnS. For C_2_H_6_, the enhancement is 0.2–0.9%, while for C_6_H_6_ the change ranges from 0 to 0.6%. These negligible conductivity modulations further validate our earlier predictions from electronic structure analyses of weak VOC−SnS interactions. The minimal conductivity response highlights major challenges for detecting these VOCs with pristine SnS monolayers.

Figure 8b demonstrates that the highest sensitivities of a SnS monolayer adsorbing C_2_H_4_, C_2_H_6_, and C_6_H_6_ are 1.7%, 1.5%, and 0.9%, respectively, with a bias voltage of 0–2.2 V. This observation provides further evidence that the SnS monolayer has low sensitivity towards VOC molecules, making it difficult to achieve effective detection.

### 3.2. Adsorption Characteristics of Pt-Decorated SnS Monolayer on VOCs

The adsorption of VOCs on the pristine SnS monolayer exhibits weak interaction and low sensitivity. Therefore, the pristine SnS monolayer would be inadequate to achieve the detection of VOCs. In this section, the adsorption and gas-sensing performance of Pt@SnS layers towards different VOCs are investigated.

After sufficient relaxation, the most stable site for Pt atom modification on the SnS monolayer surface is found to be at the H site, according to Figure 1b. Our simulations reveal strong interactions between the adsorbed Pt atom and the SnS monolayer, causing the Pt to descend in the z-direction to be level with the topmost atomic plane of SnS. Lateral displacements of the neighboring Sn atoms away from the Pt by 0.60 Å also occur due to electrostatic repulsion between the positively charged cations. Conversely, the neighboring S atoms move 0.16 Å towards the Pt because of attractive interactions. The distance between the Pt and S is measured to be 2.314 Å. The differential charge density map of the Pt-decorated SnS monolayer in Figure 1c reveals a strong charge exchange between Pt and its neighboring S atoms. Bader charge analysis further indicates a significant change in atomic charges of the modified SnS monolayer. Specifically, charge depletion occurs on two neighboring S atoms, with the electron population decreasing from 0.963e to 0.802e on one S atom, and from 0.963e to 0.840e on the other. Meanwhile, the Pt atom gains 0.332e of electronic charge. These results suggest that the modified Pt atom gains abundant electrons from the SnS monolayer, indicating a strong bond between them, consistent with the above discussion. CDD analysis of adsorbed Pt on the SnS monolayer supports this viewpoint, revealing a large amount of negative charge accumulation between Pt and its neighboring S atoms, indicating a tight bond between decorated Pt and SnS monolayer.

The band structure and DOS of the Pt-decorated SnS monolayer are presented in Figure 2b,c. The decreased bandgap of the SnS monolayer (from 1.667 eV to 1.145 eV) after Pt decoration means that it requires less energy to excite electrons from the valence band to the conduction band. The enhanced electron excitation increases the SnS monolayer’s response to changes in the gas concentration or environment. Therefore, even small amounts of VOC molecules can cause a notable change in the conductivity of the SnS monolayer. Moreover, the lower bandgap improves the speed of the electron excitation process. The electrons can easily transition from the valence band to the conduction band, facilitating rapid charge transfer and electrical conductivity changes.

The total density of states (TDOS) of the Pt-SnS monolayer shifts to the left after Pt modification, indicating increased system stability. Furthermore, highly overlapping Pt 5d orbitals with S 3p orbitals within the ranges of −4.856 eV to −3.71 eV and −0.225 eV to −0.167 eV demonstrate the formation of stable bonds between Pt and the SnS monolayer surface, allowing for stable modification on the SnS surface. Notably, a new hybridized energy level is generated due to the overlap between S 3p orbitals and Pt 5d orbitals at −0.072 eV to the left of the Fermi level, facilitating electron excitation from the valence band to the conduction band and resulting in a significant increase in conductivity of Pt@SnS. The bandgap of SnS monolayer decreases from 1.667 eV to 1.145 eV after Pt modification, indicating enhanced electron excitation.

**Figure 9:**
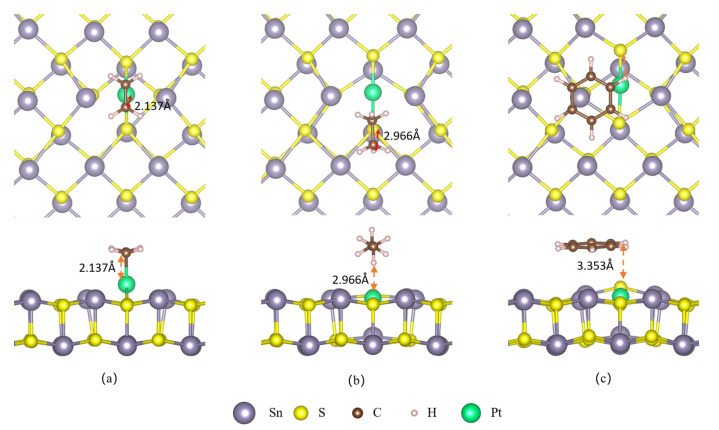
VOC adsorption on the surface of SnS monolayer. (**a**) C_2_H_4_. (**b**) C_2_H_6_. (**c**) C_6_H_6_.

The most stable configurations of adsorbed VOCs on a Pt-decorated SnS monolayer appear in Figure 9. C_2_H_4_ is found to be located at the top of the Pt atom of the SnS monolayer with its C-C bond parallel to the surface. The distance between the C atom in C_2_H_4_ and the Pt atom on the SnS monolayer surface measures 2.137 Å. Table 1 reveals that the adsorption energy of C_2_H_4_ on Pt-decorated SnS monolayer is as high as −1.503 eV. This observation suggests a strong interaction between the C_2_H_4_ and Pt-decorated SnS monolayer, which can be attributed to their close proximity and high adsorption energy. Meanwhile, C_2_H_6_ is located at the TS site with its C-C bond also parallel to the surface, and the distance between its H atom and the nearest S atom is 2.966 Å. Lastly, C_6_H_6_ is positioned above the Sn atom and shifted towards Pt, with its H atom at a distance of 3.353 Å from the nearest S atom. Comparing the intrinsic and decorated configurations, the adsorption distances of C_2_H_4_, C_2_H_6_, and C_6_H_6_ on Pt@SnS are 38.5%, 10.7%, and 4.2% lower than those on SnS (3.479, 3.322, and 3.502 Å). The shorter adsorption distances facilitate the orbital overlap and charge transfer between the gas molecules and the surface, which introduce more carriers to the surface and result in more significant changes in conductivity, thus enhancing the sensitivity of the sensor. This finding suggests that the Pt modification enhances the sensitivity of the system towards C_2_H_4_ and C_2_H_6_ gas molecules.

**Figure 10:**
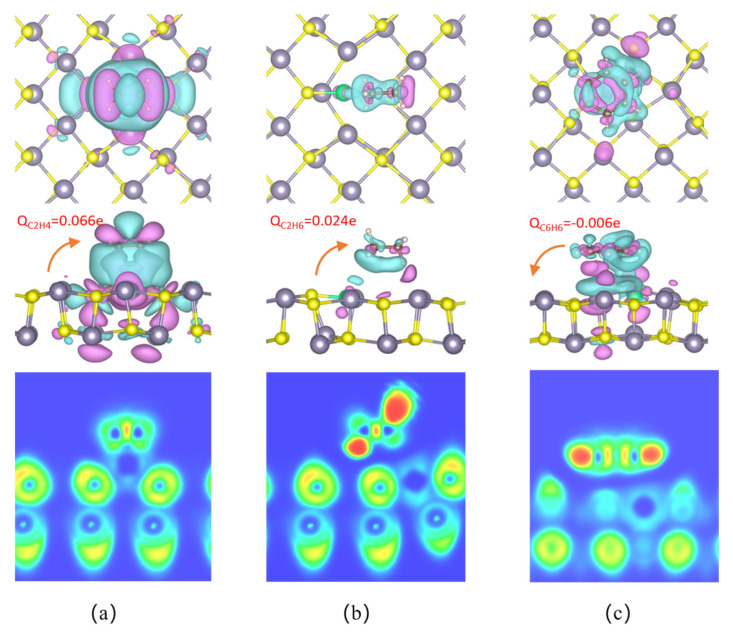
The CCD and ELF of VOC adsorption on the surface of the Pt@SnS monolayer. (**a**) C_2_H_4_. (**b**) C_2_H_6_. (**c**) C_6_H_6_.

After C_2_H_4_ adsorption on the surface of the Pt@SnS monolayer, Figure 10 reveals a connected charge accumulation region between the C and Pt atoms, while a charge depletion region is formed around the H atom. Bader analysis indicates that C_2_H_4_ as a whole gains 0.066e from the Pt@SnS monolayer surface, while Pt dopant carries a negative charge of 0.185e. Upon C_2_H_4_ adsorption, Pt dopant acts as an electron donor, extracting 0.147e of electrons from the Pt, with the SnS monolayer accepting 0.081e of these electrons. CDD analysis shows that electron depletion mainly occurs near the center of the Pt, while electron accumulation mainly occurs on the C_2_H_4_ molecule. The significant charge transfer between the C_2_H_4_ molecule and the Pt@SnS monolayer suggests that the formation of the Pt-C bond in this system is primarily governed by ionic bonding. The overlap of electron accumulation and depletion on the Pt-C bond supports this hypothesis and indicates electron hybridization during their formation process. These findings further confirm the strong interaction between the C_2_H_4_ and Pt-decorated SnS monolayer.

With C_2_H_6_ adsorption on the Pt@SnS monolayer, a charge depletion region is formed between Pt and C_2_H_6_, while a charge accumulation region is formed around the C atom. Bader charge analysis reveals that the Pt carries a negative charge of 0.383e after adsorption, indicating an additional 0.075e of electrons provided by the SnS surface, with 0.051e of electrons remaining on the Pt and 0.024e of electrons transferring to the C_2_H_6_ molecule. In other words, the Pt acts as a bridge for the charge transfer from gas molecules to the SnS monolayer, thereby promoting electron redistribution in the adsorption system.

Upon C_6_H_6_ adsorption on the Pt@SnS monolayer, a charge depletion region appears on the C atom surface facing the Pt@SnS monolayer, while a charge depletion region also appears on the surface of the Pt facing the C_6_H_6_, with side and bottom surfaces being charge accumulation regions. Bader charge analysis reveals that the C_6_H_6_ molecule as a whole loses 0.006 electrons, while the Pt carries a negative charge of 0.424e, indicating its role as an electron acceptor and gaining 0.092 electrons, with 0.086e of these electrons taken from the SnS monolayer surface.

ELF analysis reveals the absence of electron localization overlap between gas molecules C_2_H_6_ and C_6_H_6_ with SnS monolayers, implying a purely physical interaction. Conversely, the presence of discernible electron localization overlap between C_2_H_4_ and Pt suggests a robust interplay and heightened sensitivity.

**Figure 11:**
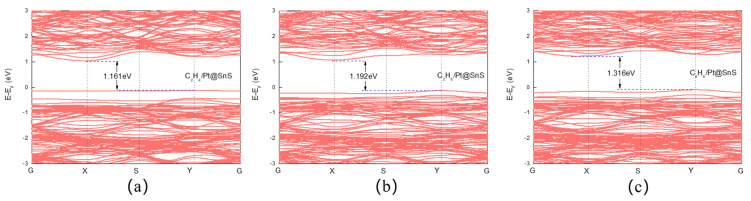
The band structures of VOC adsorption on SnS monolayers. (**a**) C_2_H_4_. (**b**) C_2_H_6_. (**c**) C_6_H_6_.

**Figure 12:**
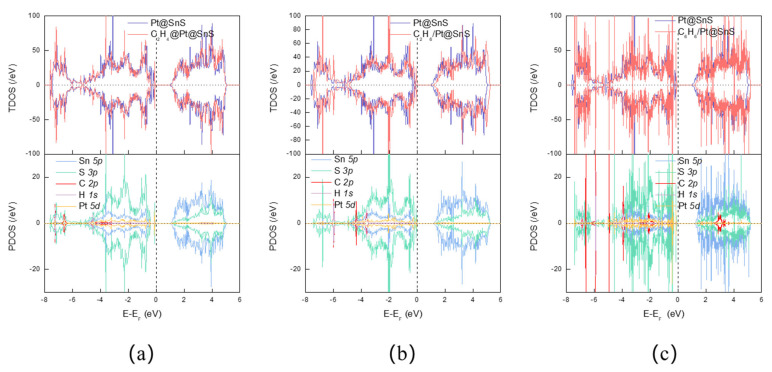
The TDOS and PDOS of VOC adsorption on SnS monolayers. (**a**) C_2_H_4_. (**b**) C_2_H_6_. (**c**) C_6_H_6_.

Figure 11 depicts that the Pt-decorated SnS monolayers undergo changes in bandgap widths upon adsorption of VOCs. Specifically, C_2_H_4_, C_2_H_6_, and C_6_H_6_ induce bandgap widths of 1.161 eV, 1.192 eV, and 1.316 eV, respectively. The degree of overlap of the density of states (DOS) provides an indirect measure of the electronic hybridization and adsorption strength among VOCs, Pt atoms, and SnS monolayers. Figure 12 shows that Pt doping expands the overlapping area of the DOS for VOCs, Pt, and SnS monolayers by increasing the overlapping area between gas molecules and substrates. Adsorbed C_2_H_4_ on Pt@SnS monolayers exhibits significant hybridization between the C-2p and S 3p orbitals at −7.254 eV, while the C 2p and S 3p, H 1s, and Pt 5d orbitals hybridize at −6.585 eV. The C 2p and Pt 5d orbitals hybridize between −5.099 eV and −2.973 eV. A steep peak appears at −3.613 eV, indicating high electron localization contributed mainly by Sn and S, with contributions from the C 2p and Pt 5d orbitals. These theoretical results demonstrate the excellent adsorption potential of Pt-decorated SnS monolayers for C_2_H_4_ gas.

Upon C_2_H_6_ adsorption, the Pt DOS overlaps with the DOS of SnS monolayer or C_2_H_6_ at −7.419 eV and −5.174 eV, −4.999 to −0.187 eV, 1.446 to 5.179 eV intervals, and −0.070 eV. A very steep hybridization peak involving Pt 5d, S 3p, and Sn 5p appears at 2.053 eV. Consequently, the Pt-decorated system exhibits higher DOS near the Fermi level than the pristine SnS monolayer after C_2_H_6_ adsorption, resulting in higher responsiveness to C_2_H_6_ gas.

In comparison to the pristine SnS monolayer, the Pt-decorated SnS monolayer exhibits a considerable rise in DOS upon C_6_H_6_ adsorption, and the hybridization peak steepens. Multiple hybridization peaks between C 2p orbitals and Sn 5p, S 3p orbitals are created above the Fermi level in the conduction band, with energies ranging from 2.511 eV to 3.638 eV. The enhanced DOS indicates that Pt@SnS with C_6_H_6_ adsorption system offers a rich variety of electronic states near the Fermi level, which facilitates the charge transfer processes upon gas adsorption. The steepening of these peaks indicates a stronger coupling and interaction between the C_6_H_6_ molecule and the Pt@SnS monolayer’s electrons. Therefore, the heightened DOS and the steepening of hybridization peaks may enhance the charge transfer and electron exchange between the adsorbed gas molecules and the Pt@SnS monolayer. This increased interaction accelerates the sensitivity of the sensor, allowing it to quickly detect in C_6_H_6_. This suggests the Pt-decorated system has a higher conductivity than that of the pristine SnS monolayer without Pt upon C_6_H_6_ adsorption.

According to Figure 7b, compared to the pristine SnS monolayer, the Pt-decorated SnS monolayer exhibits a prolonged recovery time of C_2_H_4_ desorption, reaching a value of 2.62 × 10^12^ s. This remarkable effect can be attributed to the change in the nature of adsorption upon Pt modification. In its pristine form, the SnS monolayer undergoes physical adsorption with C_2_H_4_, characterized by weak intermolecular interactions, enabling easy dissociation and departure of C_2_H_4_ molecules from the SnS monolayer surface. However, after the introduction of Pt decoration, the Pt@SnS monolayer establishes a chemical adsorption interface with C_2_H_4_, exhibiting a significantly elevated adsorption energy of −1.503 eV. The intensified interaction between C_2_H_4_ molecules and the Pt-decorated SnS monolayer surface leads to an extended recovery period upon gas removal. This can be attributed to the presence of a higher binding energy, establishing a substantial thermodynamic barrier that impedes the desorption of gas molecules from the surface. However, an increase in temperature to 498 K remarkably reduces the recovery time to 162.33 s, demonstrating the feasibility of repeated utilization through effective detachment of C_2_H_4_ from the Pt@SnS surface.

In contrast, our calculations predict fast room temperature recovery times of 1.37 × 10^−7^ s and 9.60 × 10^−6^ s for C_2_H_6_ and C_6_H_6_ on Pt-functionalized SnS monolayers, respectively. This rapid reversibility stems from the physisorption of these VOCs, with weaker adsorption energies of just −0.363 eV for C_2_H_6_ and −0.472 eV for C_6_H_6_. The minimal physical binding means only small amounts of thermal energy are required to detach the VOC molecules when gas exposure ceases. Thus, the Pt-SnS system can quickly revert to its unperturbed baseline state as the VOCs desorb, enabling exceptional sensor resettability and cycling.

**Figure 13:**
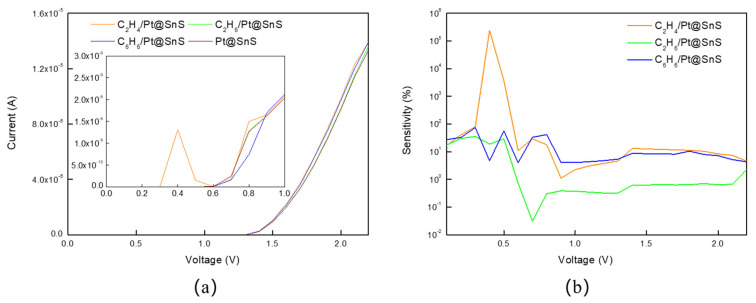
The (**a**) current−voltage curves and (**b**) sensitivity of VOC adsorption on Pt@SnS monolayer.

As plotted in Figure 13a, a sharp peak in the current is observed at 0.4 V for Pt@SnS upon adsorption of C_2_H_4_. The current curves of Pt@SnS with adsorbed C_2_H_6_ or C_6_H_6_ can also be clearly distinguished from that of pristine Pt@SnS without adsorbed VOCs. According to the I–V curves in Figure 8a and Figure 13a, the electrical conductance of Pt@SnS for C_2_H_4_ was 1.88 to 50623.3 times higher than that of SnS under a voltage range of 0.1–2.2 V, while the electrical conductance of Pt@SnS for C_2_H_6_ and C_6_H_6_ was 1.60 to 21.0 and 1.72 to 19.79 times higher than that of SnS, respectively. This is consistent with the previous description that the Pt decoration system has better electrical conductivity than intrinsic SnS.

Figure 13b demonstrates that the sensitivity of Pt-decorated SnS monolayers towards C_2_H_4_, C_2_H_6_, and C_6_H_6_ reaches peak values of 241,921.7%, 35.7%, and 74.3%, respectively, at bias voltages of 0.4 V, 0.3 V, and 0.3 V, respectively. These values are 142,306.9, 23.8, and 82.6 times higher than those of pristine SnS monolayers. Apart from the response peak at 0.3V, Pt@SnS adsorbing C_6_H_6_ shows two additional peaks at 0.5 V and 0.3 V with sensitivities of 56.6% and 33.2% respectively. By identifying these three response peaks, it is possible to discriminate C_6_H_6_ gas molecules from a variety of gases.

This finding highlights that Pt decoration significantly enhances the sensitivity of the SnS monolayer towards VOC molecules, making it a promising sensing material for VOC detection. Moreover, the significantly higher sensitivity of the Pt-decorated SnS monolayer towards C_2_H_4_ compared to other gases is consistent with its higher adsorption energy and charge transfer towards C_2_H_4_, as discussed earlier. Therefore, the Pt-decorated SnS monolayer exhibits a high selectivity towards C_2_H_4_ detection.

In this study, we conducted a comparative analysis of pristine SnS monolayers and those decorated with Pt, in relation to previously reported VOC sensors (Table S1) [29,30,31,32,33,34,35,36,37,38,39,40,41,42]. Our results demonstrate that SnS monolayers exhibit significantly greater sensitivity to VOCs than other VOC sensors reported. Specifically, SnS monolayers exhibit sensitivities approximately 51.3 and 4300 times greater than Nb-doped PtS_2_ [29] and Au-doped MoS_2_ [30], respectively, when used as C_2_H_4_ sensors. Furthermore, Pt-decorated SnS monolayers show sensitivity to C_6_H_6_ about 7.5 times greater than graphene-like ZnO [31]. Additionally, SnS monolayers exhibit high reusability when sensing VOCs. With high sensitivity to C_2_H_4_, Pt-decorated SnS monolayers can recover in 162.33 s at 498 K. In contrast, Nb-doped PtS_2_ exhibits a slow recovery time of 2.09 × 10^7^ s at 498 K, which limits its utility to a disposable sensor. Compared to other 2D materials, Pt-SnS monolayers exhibit moderately fast room temperature recovery for C_2_H_6_ and C_6_H_6_ adsorption, as highlighted in Table S1. The predicted 1.37 × 10^−7^ s C_2_H_6_ recovery time is slower than ultrafast values for Au-MoS_2_ (4.13 × 10^−9^ s) and InP_3_ (1.88 × 10^−8^ s) [35] yet orders of magnitude faster than WO_3_ (7.89 s) and oxygen-terminated defective surface decorate WO_3_ (7.35 × 10^4^ s) [32]. For C_6_H_6_, the 9.60 × 10^−6^ s Pt-SnS recovery exceeds stanene (2.25 × 10^−12^ s) [38] and SiC (−0.28 ns) [41] but is considerably swifter than Al_2_C (8.58 × 10^24^ s) [33], InP_3_ (514.32 s) [35], Al@MnO_2_ (7.23 × 10^27^ s) [39], and Al@C_2_N (9.43 × 10^13^ s) [40]. This balanced combination of sensitivity and feasible resettability makes Pt-SnS a promising room temperature VOC sensing candidate.

## 4. Conclusions

The sensing capabilities of pristine and Pt-decorated SnS monolayers towards VOCs were investigated using DFT calculations. Pt decoration introduces new energy levels at the top of valence bands, which enhances the overlap region in the density of states between VOCs, SnS, and Pt. Charge analysis indicates that Pt promotes electron redistribution in the adsorption system, leading to a substantial improvement in the sensing performance. Pt-decorated SnS monolayers exhibited a sensitivity of 241,921.7%, 35.7%, 74.3% towards C_2_H_4_, C_2_H_6_, and C_6_H_6_, respectively, which is 142,306.9, 23.8, 82.6 times that of a pristine SnS monolayer, exhibited excellent sensitivity for sensing VOCs, as well as high selectivity for C_2_H_4_ detection. The recovery time of Pt-decorated SnS monolayers towards these gases at room temperature was also comparable. The Pt-decorated SnS monolayer demonstrated favorable recovery times of 1.37 × 10^−7^ s and 9.60 × 10^−6^ s for C_2_H_6_ and C_6_H_6_ at room temperature. Although Pt-decorated SnS monolayers exhibited prolonged recovery times at 298 K, heating them to 498 K resulted in a decrease in recovery time to 162.33 s. This confirms that Pt-decorated SnS monolayers can be reused as VOC sensors. The findings of this research demonstrate the potential of SnS monolayers as highly selective, sensitive, and reversible VOC sensing materials.

## Figures and Tables

**Table 1:** The list of adsorption energy (E*_ad_*), adsorption distance (d), charge transfer (ΔQ), and energy gaps (E_g_) between adsorbate and SnS monolayer.

VOCs	E*_ad_* (eV)	d (Å) ^1^	ΔQ (e)	E_g_ (eV)
C_2_H_4_/SnS	−0.532	3.479	0.012	1.570
C_2_H_6_/SnS	−0.314	3.322	0.016	1.611
C_6_H_6_/SnS	−0.601	3.502	0.011	1.565
C_2_H_4_/Pt@SnS	−1.503	2.137	0.066	1.161
C_2_H_6_/Pt@SnS	−0.363	2.966	0.024	1.192
C_6_H_6_/Pt@SnS	−0.472	3.353	−0.006	1.316

^1^ Adsorption distance is defined as the distance between the adsorbate and the closest atom in the substrate.

## Data Availability

Not applicable.

## Supplementary Materials

The following supporting information can be downloaded at: https://www.mdpi.com/article/10.3390/s23177319/s1, Figure S1: The two-probe simulation model for VOCs sensing; Table S1: Comparison between this work with previous reports.
