# Supplementary material for: A DFT Study of Volatile Organic Compounds Detection on Pristine and Pt-Decorated SnS Monolayers
Source: Sensors (Basel). 2023 Aug 22;23(17):7319. doi: 10.3390/s23177319 (PMC10490194; doi:10.3390/s23177319)
Supplement: Supplementary file 1 [file sensors-23-07319-s001.zip › sensors-2556844-supplementary.docx]

**A DFT Study of Volatile Organic Compounds Detection on Pristine and Pt-Decorated SnS Monolayers**

Jiayin Wu ^1^, Zhongbao Li ^2^, Aiping Luo ^1^, and Xiaobo Xing ^3,^*

^1^ School of Information and Optoelectronic Science and Engineering, South China Normal University,
Guangzhou 510006, China, wujiayin@m.scnu.edu.cn, luoaiping@scnu.edu.cn

^2^ School of Materials and Chemistry, Tongren University, Tongren 554300, China; zongbaoli1982@163.com

^3^ South China Academy of Advanced Optoelectronics, South China Normal University, Guangzhou 510006; [xingxiaobo@scnu.edu.cn](mailto:xingxiaobo@scnu.edu.cn)

* Correspondence: xingxiaobo@scnu.edu.cn


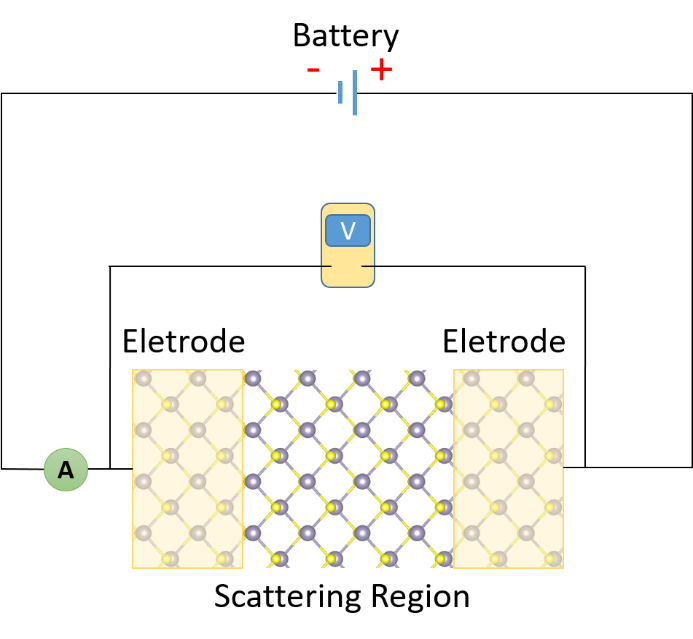


**Figure S1.** The two-probe simulation model for VOCs sensing.

Table S1

Comparison between this work with previous reports

| Sensing material | Gas | Adsorption energy E_ad_(eV) | Recovery time, temperature | Sensitivity | Ref |
| --- | --- | --- | --- | --- | --- |
| *SnS* | C_2_H_4_ | -0.532 | 9.59×10^-5^ s, 298K | 1.7% | This work |
| *SnS* | C_2_H_6_ | -0.314 | 2.04×10^-8^ s, 298K | 1.5% | This work |
| *SnS* | C_6_H_6_ | -0.601 | 1.46×10^-3^ s, 298K | 0.9% | This work |
| *Pt@SnS* | C_2_H_4_ | -1.503 | 162.33 s, 498K | 241921.7% | This work |
| *Pt@SnS* | C_2_H_6_ | -0.363 | 1.37×10^-7^ s, 298K | 35.7% | This work |
| *Pt@SnS* | C_6_H_6_ | -0.472 | 9.60×10^-6^ s, 298K | 74.3% | This work |
| *Nb-doped PtS_2_* | C_2_H_4_ | -1.91 | 1.95×10^20^ s, 298K  2.09×10^7^ s, 498K | 4717.4% | [1] |
| *Au-doped MoS_2_* | C_2_H_4_ | -0.410 | 8.58×10^-7^s,298K* | 56.26% | [2] |
| *Au-doped MoS_2_* | C_2_H_6_ | -0.273 | 4.13×10^-9^s,298K* | 81.60% | [2] |
| *graphene-like ZnO* | C_6_H_6_ | NR | NR | 9.93%** | [3] |
| *WO_3_* | C_2_H_6_ | NR | 7.89s,300K  5.51×10^-5^ s,500K | NR | [4] |
| *OS-WO_3_* | C_2_H_6_ | NR | 1.52×10^-6^ s,300K  5.12×10^-9^ s,500K | NR | [4] |
| *ODS-WO_3_* | C_2_H_6_ | NR | 7.35×10^4^ s,300K  1.32×10^-2^ s,500K | NR | [4] |
| *Al_2_C* | C_6_H_6_ | -2.243 | 8.58×10^24^s,298K* | NR | [5] |
| *GO* | C_2_H_4_ | −0.36 | 1.22×10^-7^s,298K* | NR | [6] |
| *Y doped GO* | C_2_H_4_ | −2.22 | 3.50×10^24^s,298K* | NR | [6] |
| *InP_3_* | C_2_H_4_ | -0.7488 | 0.46s,298K* | NR | [7] |
| *InP_3_* | C_2_H_6_ | -0.3118 | 1.88×10^-8^s,298K* | NR | [7] |
| *InP_3_* | C_6_H_6_ | -0.9290 | 514.32s,298K* | NR | [7] |
| *χ3-borophene* | C_2_H_4_ | 1.14 | 5.25×10^-33^s,298K* | NR | [8] |
| *χ3-borophene* | C_2_H_6_ | 0.27 | 2.72×10^-18^s,298K* | NR | [8] |
| *χ3-borophene* | C_6_H_6_ | 0.62 | 3.27×10^-24^s,298K* | NR | [8] |
| *NiO@SnS_2_* | C_2_H_4_ | -0.986 | 289.767 s,343K  4.189 s,393K | NR | [9] |
| *CuO@SnS_2_* | C_2_H_4_ | -1.157 | 9.626×10^4^s,343K  664.812 s,393K | NR | [9] |
| *stanene* | C_6_H_6_ | -0.08 | 2.25×10^-12^s,298K* | NR | [10] |
| *MnO_2_* | C_6_H_6_ | -0.514 | 4.1×10^-5^s,298K* | NR | [11] |
| *Al@MnO_2_* | C_6_H_6_ | -2.416 | 7.23×10^27^s,298K* | NR | [11] |
| *C_2_N* | C_6_H_6_ | -0.556 | 2.53×10^-4^s,298K* | NR | [12] |
| *Al@C_2_N* | C_6_H_6_ | -1.595 | 9.43×10^13^s,298K* | NR | [12] |
| *SiC* | C_6_H_6_ | NR | -0.28 ns | NR | [13] |
| *SnS_2_* | C_2_H_4_ | 0.10 | 2.04×10^-15^s,298K* | NR | [14] |
| *Ru_3_@SnS_2_* | C_2_H_4_ | 0.88 | 1.31×10^-28^s,298K* | NR | [14] |

NR The data is no report from the paper.

*This data was not directly reported in the referenced publication, but rather derived from Equation 4 utilizing the adsorption energy in the referenced publication.

**This data was extracted from the graph of the paper

1. Yao, W.; Guan, H.; Zhang, K.; Wang, G.; Wu, X.; Jia, Z. Nb-Doped PtS2 Monolayer for Detection of C2H2 and C2H4 in on-Load Tap-Changer of the Oil-Immersed Transformers: A First-Principles Study. *Chem. Phys. Lett.* **2022**, *802*, 139755, doi:10.1016/j.cplett.2022.139755.

2. Jiang, T.; He, Q.; Bi, M.; Chen, X.; Sun, H.; Tao, L. First-Principles Calculations of Adsorption Sensitivity of Au-Doped MoS2 Gas Sensor to Main Characteristic Gases in Oil. *J. Mater. Sci.* **2021**, *56*, 13673–13683, doi:10.1007/s10853-021-06168-7.

3. Harun Achmad, M.; Azhar Mansoor Al Sarraf, A.; Bokov, D.O.; Raya, I.; Derakhshandeh, M. A DFT Study on the Ag-Decorated ZnO Graphene-like Nanosheet as a Chemical Sensor for Ethanol: Explaining the Experimental Observations. *Inorg. Chem. Commun.* **2021**, *133*, 108892, doi:10.1016/j.inoche.2021.108892.

4. Li, J.-H.; Wu, J.; Yu, Y.-X. DFT Exploration of Sensor Performances of Two-Dimensional WO3 to Ten Small Gases in Terms of Work Function and Band Gap Changes and I-V Responses. *Appl. Surf. Sci.* **2021**, *546*, 149104, doi:10.1016/j.apsusc.2021.149104.

5. Rahimi, R.; Solimannejad, M.; Chaudhari, A. Toxic Volatile Organic Compounds Sensing by Al2C Monolayer: A First-Principles Outlook. *J. Hazard. Mater.* **2021**, *403*, 123600, doi:10.1016/j.jhazmat.2020.123600.

6. Tabari, L.; Farmanzadeh, D. Yttrium Doped Graphene Oxide as a New Adsorbent for H2O, CO, and Ethylene Molecules: Dispersion-Corrected DFT Calculations. *Appl. Surf. Sci.* **2020**, *500*, 144029, doi:10.1016/j.apsusc.2019.144029.

7. Yang, H.; Wang, Z.; Ye, H.; Zhang, K.; Chen, X.; Zhang, G. Promoting Sensitivity and Selectivity of HCHO Sensor Based on Strained InP3 Monolayer: A DFT Study. *Appl. Surf. Sci.* **2018**, *459*, 554–561, doi:10.1016/j.apsusc.2018.08.014.

8. Li, W.; Jiang, Q.; Li, D.; Ao, Z.; An, T. Density Functional Theory Investigation on Selective Adsorption of VOCs on Borophene. *Chin. Chem. Lett.* **2021**, *32*, 2803–2806, doi:10.1016/j.cclet.2021.01.026.

9. Chen, Y.; Gui, Y.; Chen, X. Adsorption and Gas-Sensing Properties of C2H4, CH4, H2, H2O on Metal Oxides (CuO, NiO) Modified SnS2 Monolayer: A DFT Study. *Results Phys.* **2021**, *28*, 104680, doi:10.1016/j.rinp.2021.104680.

10. Li, Y.; Yu, C.-M. DFT Study of the Adsorption of C6H6 and C6H5OH Molecules on Stanene Nanosheets: Applications to Sensor Devices. *Phys. E Low-Dimens. Syst. Nanostructures* **2021**, *127*, 114533, doi:10.1016/j.physe.2020.114533.

11. Cao, J.; Wu, F.; Wen, M.; Peng, J.; Yang, Y.; Dong, H. Adsorption Mechanism of Typical VOCs on Pristine and Al-Modified MnO2 Monolayer. *Appl. Surf. Sci.* **2021**, *539*, 148164, doi:10.1016/j.apsusc.2020.148164.

12. Su, Y.; Ao, Z.; Ji, Y.; Li, G.; An, T. Adsorption Mechanisms of Different Volatile Organic Compounds onto Pristine C2N and Al-Doped C2N Monolayer: A DFT Investigation. *Appl. Surf. Sci.* **2018**, *450*, 484–491, doi:10.1016/j.apsusc.2018.04.157.

13. Yadav, A. Monolayer Silicon Carbide as an Efficient Adsorbent for Volatile Organic Compounds: An Ab Initio Approach. *Silicon* **2023**, *15*, 1563–1569, doi:10.1007/s12633-022-02120-9.

14. Peng, R.; Zeng, W.; Zhou, Q. Adsorption and Gas Sensing of Dissolved Gases in Transformer Oil onto Ru3-Modified SnS2: A DFT Study. *Appl. Surf. Sci.* **2023**, *615*, 156445, doi:10.1016/j.apsusc.2023.156445.
